# Supplementary material for: Lingering single-strand breaks trigger Rad51-independent homology-directed repair of collapsed replication forks in the polynucleotide kinase/phosphatase mutant of fission yeast
Source: PLoS Genet. 2017 Sep 18;13(9):e1007013. doi: 10.1371/journal.pgen.1007013 (PMC5626526; doi:10.1371/journal.pgen.1007013)
Supplement: S1 Table — (DOCX) [file pgen.1007013.s001.docx]

**Table S1.** *S. pombe* strains used in this study

| **Strain** | **Genotype** | | **Source** |
| --- | --- | --- | --- |
| AS5121 | *h^-^ leu1-32* | | Lab stock |
| AS5122 | *h^-^ leu1-32 brc1::HphMX6* | | Lab stock |
| JW4673 | *h^+^ leu1-32 brc1-wt-2GFP::HphMX6* | | Lab stock |
| JW4675 | *h^+^ leu1-32 brc1-T672A-2GFP::HphMX6* | | Lab stock |
| JW4700 | *h^+^ leu1-32 brc1-K710M-2GFP::HphMX6* | | Lab stock |
| AS5303 | *h^-^ leu1-32 pnk1::KanMX6* | | Lab stock |
| AS5304 | *h^+^ leu1-32 brc1::HphMX6 pnk1::KanMX6* | | Lab stock |
| AS5396 | *h^+^ leu1-32 pnk1::kanMX6 brc1-wt-2GFP::HphMX6* | | This study |
| AS5397 | *h^+^ leu1-32 pnk1::kanMX6 brc1-T672A-2GFP::HphMX6* | | This study |
| AS5398 | *h^+^ leu1-32 pnk1::kanMX6 brc1-K710M-2GFP::HphMX6* | | This study |
| AS5399 | *h^-^ leu1-32 pnk1::KanMX6* | | This study |
| KS1452 | *h^-^ leu1-32 ura4-D18 ade6-704 chk1::ura4^+^* | | Lab stock |
| AS5400 | *h^-^ leu1-32 ura4-D18 brc1::HphMX6 pnk1::KanMX6* | | This study |
| AS5401 | *h^-^ leu1-32 ura4-D18 ade6-704 brc1::HphMX6 chk1::ura4^+^* | | This study |
| AS5402 | *h^-^ leu1-32 ura4-D18 ade6-704 pnk1::KanMX6 chk1::ura4^+^* | | This study |
| AS5403 | *h^+^ leu1-32 ura4-D18 ade6-704 brc1::HphMX6 pnk1::KanMX6 chk1::ura4^+^* | | This study |
| TM2937 | *h^-^ leu1-32 ura4-D18 ade6-M216 his3-D1 rad3::ura4^+^* | | Lab stock |
| AS5404 | *h^-^ leu1-32 ura4-D18 pnk1::KanMX6 rad3::ura4^+^* | | This study |
| AS5405 | *h^-^ leu1-32 ura4-D18 brc1::HphMX6 rad3::ura4^+^* | | This study |
| AS5406 | *h^-^ leu1-32 ura4-D18 brc1::HphMX6 pnk1::KanMX6 rad3::ura4^+^* | | This study |
| AS5407 | *h^+^ leu1-32 ura4-D18 Rad52-YFP:KanMX6* | | This study |
| AS5290 | *h^+^ leu1-32 ura4-D18 brc1::HphMX6 Rad52-YFP:KanMX6* | | Lab stock |
| AS5408 | *h^-^ leu1-32 pnk1::KanMX6 Rad52-YFP:KanMX6* | | This study |
| AS5409 | *h^+^ leu1-32 brc1::HphMX6 pnk1::KanMX6 Rad52-YFP:KanMX6* | | This study |
| LLD3427 | *h^-^ ura4-D18 leu1-32 chk1-9myc-2HA6His::ura4^+^* | | Lab stock |
| AS5414 | *h^-^ leu1-32 ura4-D18 pnk1::KanMX6 chk1-9myc-2HA6His::ura4* | | This study |
| AS5415 | *h^+^ leu1-32 ura4-D18* | | This study |
| AS5411 | *h^-^ leu1-32 ura4-D18 pnk1::KanMX6* | | This study |
| YYY4320 | *h^-^ leu1-32 ura4-D18 mre11::KanMX6* | | Lab stock |
| AS5416 | *h+ leu1-32 ura4-D18 pku80::HphMX6* | | This study |
| AS5417 | *h^-^ leu1-32 ura4-D18 pnk1::KanMX6 mre11::NatMX6* | | This study |
| AS5418 | *h^-^ leu1-32 ura4-D18 pnk1::KanMX6 pku80::HphMX6* | | This study |
| OL4178 | *h^-^ leu1-32 ura4-D18 pku80::HphMX6 mre11::KanMX6* | | Lab stock |
| AS5419 | *h^-^ leu1-32 ura4-D18 pnk1::KanMX6 pku80::HphMX6 mre11::NatMX6* | | This study |
| OL4590 | *h^-^ leu1-32 ura4-D18 ctp1::HphMx6* | | Lab stock |
| OL4175 | *h^-^ leu1-32 ura4-D18 (294) exo1::ura4^+^* | | Lab stock |
| AS5420 | *h^-^ leu1-32 ura4-D18 pnk1::KanMX6 ctp1::NatMX6* | | This study |
| AS5421 | *h^-^ leu1-32 ura4-D18 (or 294) pnk1::KanMX6 exo1::ura4^+^* | | This study |
| OL4130 | *h^+^ leu1-32 ura4-D18 pku80::HphMX6 ctp1::NatMX6* | | Lab stock |
| OL4132 | *h^+^ leu1-32 ura4-D18 (or 294) his4-239 ctp1::natMX6 exo1::ura4^+^* | | Lab stock |
| AS5330 | *h^-^ ura4-D18 leu1-32 pku80::KanMX6 exo1::ura4^+^* | | Lab stock |
| OL4131 | *h^+^ leu1-32 ura4-D18 (or 294) pku80::HphMX6 ctp1::NatMX6 exo1::ura4^+^* | | Lab stock |
| AS5422 | *h^+^ leu1-32 ura4-D18 pnk1::KanMX6 pku80::HphMX6 ctp1::NatMX6* | | This study |
| AS5423 | *h^+^ leu1-32 ura4-D18 (or 294) pnk1::KanMX6 ctp1::NatMX6 exo1::ura4^+^* | | This study |
| AS5424 | *h^+^ leu1-32 ura4-D18 (or 294) pnk1::KanMX6 pku80::HphMX6 exo1::ura4^+^* | | This study |
| AS5425 | *h^-^ leu1-32 ura4-D18 (or 294) pnk1::KanMX6 pku80::HphMX6 ctp1::NatMX6 exo1::ura4^+^* | | This study |
| PS2383 | *h^-^ smt0 leu1-32 ura4-D18 rhp51::ura4^+^* | | Lab stock |
| AS5427 | *h^+^ leu1-32 ura4-D18 pnk1::KanMX6 rhp51::ura4^+^* | | This study |
| AS5428 | *h^+^ leu1-32 ura4-D18 pnk1::KanMX6* | | This study |
| PS2382 | *h^-^ smt0 leu1-32 ura4-D18 rhp54::ura4^+^* | | Lab stock |
| AS5429 | *h^-^ leu1-32 ura4-D18 pnk1::KanMX6 rhp54::ura4^+^* | | This study |
| TMN3317 | *h^+^ leu1-32 ura4-D18 ade6-M216 his3-D1 rad52-D2::LEU2* | | Lab stock |
| PR109 | *h^-^* | Lab stock | |
| OL5559 | *h^+^ leu1-32 ura4-D18 pnk1::KanMX6 pDblet:rad52^+^* | | This study |
| OL5560 | *h^-^ smt0 leu1-32 ura4-D18 rad52-D2::LEU2 pDblet:rad52^+^* | | This study |
| OL5561 | *h^-^ smt0 leu1-32 ura4-D18 rad52-D2::LEU2 pnk1::KanMX6 pDblet:rad52^+^* | | This study |
| NR2839 | *h^+^ leu1-32 ura4-D18 ade6-L469/pUC8/his3/ade6-M375 his3-D1 arg3-D1* | | Lab stock |
| AS5432 | *h^-^ leu1-32 ura4-D18 ade6-L469/pUC8/his3/ade6-M375 his3-D1 pnk1::KanMX6* | | This study |
| AS5433 | *h^+^ leu1-32 ura4-D18 ade6-L469/pUC8/his3/ade6-M375 his3-D1 brc1::HphMX6* | | This study |
| AS5434 | *h^-^ leu1-32 ura4-D18 ade6-L469/pUC8/his3/ade6-M375 his3-D1 brc1::HphMX6 pnk1::KanMX6* | | This study |
| CD4692 | *h^+^ ura4-D18 leu1-32 mus81::NatMX6* | | Lab stock |
| AS5435 | *h^+^ leu1-32 ura4-D18 pnk1::KanMX6 mus81::NatMX6* | | This study |
| PH3687 | *h^-^ leu1-32 ura4-D18 eme1::ura4^+^* | | Lab stock |
| AS5436 | *h^-^ leu1-32 ura4-D18 pnk1::KanMX6 eme1::ura4^+^* | | This study |
| NB3155 | *h^+^ leu1-32 ura4-D18 rqh1::ura4^+^* | | Lab stock |
| AS5437 | *h^-^ leu1-32 slx1::NatMX6* | | This study |
| AS5438 | *h^-^ leu1-32 ura4-D18 brc1::HphMX6 slx1::NatMX6* | | This study |
| AS5439 | *h^+^ leu1-32 pnk1::KanMX6 slx1::NatMX6* | | This study |
| AS5440 | *h^+^ leu1-32 brc1::HphMX6 pnk1::KanMX6 slx1::NatMX6* | | This study |
| YYY4247 | *h^-^ leu1-32 ura4-D18 swi10::ura4^+^* | | Lab stock |
| AS5441 | *h^-^ leu1-32 ura4-D18 brc1::HphMX6 swi10::ura4^+^* | | This study |
| AS5442 | *h^-^ leu1-32 ura4-D18 pnk1::KanMX6 swi10::ura4^+^* | | This study |
| AS5443 | *h^+^ leu1-32 ura4-D18 brc1::HphMX6 pnk1::KanMX6 swi10::ura4^+^* | | This study |
| EM4889 | *h^+^ leu1-32 ura4-D18 rad11-GFP:HphMX6* | | Lab stock |
| AS5285 | *h^+^ leu1-32 ura4-D18 brc1::HphMX6 rad11-GFP::HphMX6* | | Lab stock |
| AS5449 | *h^-^ leu1-32 ura4-D18 pnk1::KanMX6 rad11-GFP::HphMX6* | | This study |
| AS5450 | *h^-^ leu1-32 ura4-D18 pnk1::KanMX6 brc1::HphMX6 rad11-GFP::HphMX6* | | This study |
| OM810 | *h^-^ leu1-32* | | Lab stock |
| MG5552 | *h^-^ leu1-32 ura4-D18 pnk1::ura4^+^* | | This study |
| MG5553 | *h^?^ leu1-32 cds1::KanMX6* | | This study |
| MG5554 | *h^?^ leu1-32 chk1::HphMX6* | | Lab stock |
| MG5555 | *h^?^ leu1-32 cds1::KanMX6 chk1::HphMX6* | | Lab stock |
| MG5556 | *h^?^ leu1-32 ura4-D18 pnk1::ura4^+^ cds1::KanMX6* | | This study |
| MG5557 | *h^?^ leu1-32 ura4-D18 pnk1::ura4^+^ chk1::HphMX6* | | This study |
| MG5558 | *h^?^ leu1-32 ura4-D18 pnk1::ura4^+^ cds1::KanMX6 chk1::HphMX6* | |  |
